# Diacylglycerol kinase β promotes dendritic outgrowth and spine maturation in developing hippocampal neurons

**DOI:** 10.1186/1471-2202-10-99

**Published:** 2009-08-19

**Authors:** Yasukazu Hozumi, Masahiko Watanabe, Koichi Otani, Kaoru Goto

**Affiliations:** 1 Department of Anatomy and Cell Biology, Yamagata University School of Medicine, Yamagata 990-9585, Japan; 2 Department of Anatomy, Hokkaido University School of Medicine, Sapporo 060-8638, Japan; 3 Department of Psychiatry, Yamagata University School of Medicine, Yamagata 990-9585, Japan

## Abstract

**Background:**

Diacylglycerol kinase (DGK) is an enzyme that phosphorylates diacylglycerol to phosphatidic acid and comprises multiple isozymes of distinct properties. Of DGKs, mRNA signal for DGKβ is strongly detected in the striatum, and one of the transcripts derived from the human DGKβ locus is annotated in GenBank as being differentially expressed in bipolar disorder patients. Recently, we have reported that DGKβ is expressed in medium spiny neurons of the striatum and is highly concentrated at the perisynapse of dendritic spines. However, it remains elusive how DGKβ is implicated in pathophysiological role in neurons at the cellular level.

**Results:**

In the present study, we investigated the expression and subcellular localization of DGKβ in the hippocampus, together with its functional implication using transfected hippocampal neurons. DGKβ is expressed not only in projection neurons but also in interneurons and is concentrated at perisynaptic sites of asymmetrical synapses. Overexpression of wild-type DGKβ promotes dendrite outgrowth at 7 d in *vitro *(DIV) and spine maturation at 14 DIV in transfected hippocampal neurons, although its kinase-dead mutant has no effect.

**Conclusion:**

In the hippocampus, DGKβ is expressed in both projection neurons and interneurons and is accumulated at the perisynapse of dendritic spines in asymmetrical synapses. Transfection experiments suggest that DGKβ may be involved in the molecular machineries of dendrite outgrowth and spinogenesis through its kinase activity.

## Background

Following activation of Gq protein-coupled receptors in response to external stimuli, phospholipase C (PLC) yields a pair of second messengers, diacylglycerol (DG) and inositol 1,4,5-trisphosphate [1,2]. In this system, diacylglycerol kinase (DGK) phosphorylates DG to produce another second messenger, phosphatidic acid (PA). One of the best known functional roles of DGK is in the regulation of protein kinase C (PKC), for which DG acts as an allosteric activator, and whose activity plays a central role in many different cell types [3-5]. In addition, recent studies have revealed that PA also acts as a messenger to regulate a number of signaling molecules [6,7]. Therefore DGK is thought to mediate signal transduction by modulating levels of DG and PA, i.e. the attenuation of DG and the production of PA.

To date, ten DGK isozymes have been identified from mammalian cells [8-10]. Of DGKs, DGKβ is shown to be expressed abundantly in the striatum, accumbens nucleus, olfactory bulb, and hippocampus, the areas that correspond to dopaminergic projection fields, and its expression level increases in developing brain [11-13]. Analysis of human DGKβ gene reveals the existence of a total of 16 different splice variants, one of which corresponds to an EST annotated in GenBank as differentially expressed in bipolar disorder patients [14]. This suggests that alteration of the expression, localization, and/or activity of this isozyme might result in synaptic imbalance and altered neuronal excitability in this field, which could lead to mood disorders. At the cellular level, we have recently shown that DGKβ is selectively expressed in medium spiny neurons (MSNs) of the striatum and enriched in the perisynaptic site at corticostriatal and thalamostriatal synapses [13]. In addition, experimental analysis in transfected cells reveals that overexpression of DGKβ causes altered assembly of actin stress fibers while a kinase-dead mutant of DGKβ abolishes its colocalization with stress fibers, suggesting that the enzymatic activity of DGKβ may be involved in actin filament assembly [15]. Because the actin cytoskeleton is thought to have important roles in regulating morphological changes of dendritic spines [16,17], it is suggested that DGKβ plays a role in controlling the shape of dendritic spines in neurons. However, it remains elusive how DGKβ is implicated in pathophysiological roles in neurons.

To gain an insight in the functional implication of DGKβ, we examined its detailed localization and the functional properties in hippocampal neurons. In this study, we performed the high-resolution immunohistochemical study together with the transfection of wild-type DGKβ and its kinase-dead mutant into primary cultured hippocampal neurons. Here, we show that DGKβ is predominantly localized to perisynaptic membrane of hippocampal neurons and induces dendrite outgrowth and spine maturation in developing neurons through its enzymatic activity.

## Results

In previous studies we have shown that gene of DGKβ is highly expressed in the striatum, accumbens nucleus, hippocampus, olfactory tubercle, and olfactory bulb [11] and that in the striatum DGKβ is selectively expressed in MSNs and highly enriched in the perisynaptic site at corticostriatal and thalamostriatal synapses [13]. In the present study, we investigated the expression and subcellular localization of DGKβ in the hippocampus, together with its functional implication using transfected hippocampal neurons.

### General distribution in the hippocampus

**Figure 1:**
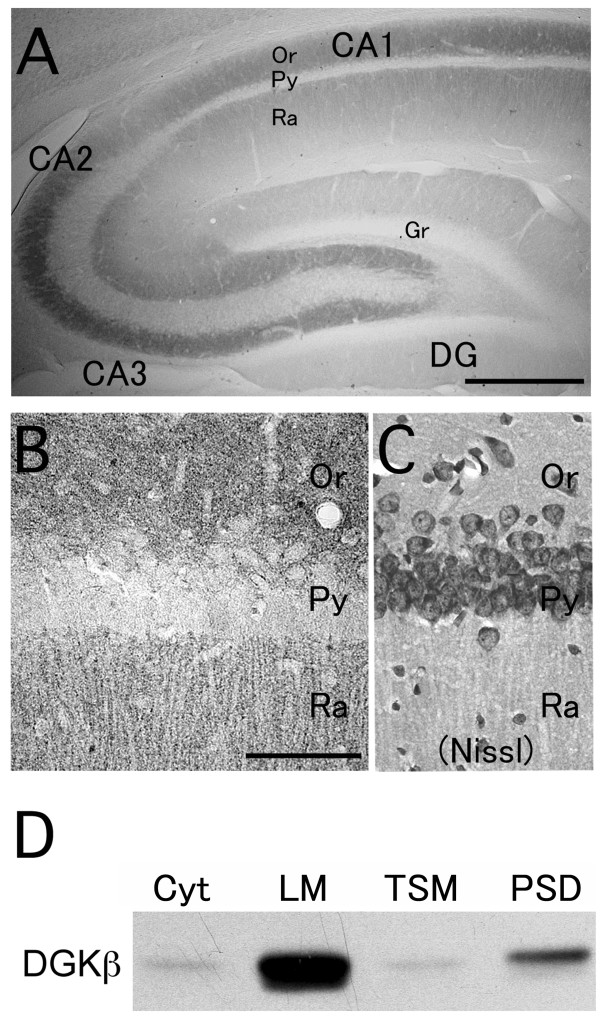
**Immunohistochemistry and immunoblot analysis showing DGKβ distribution in the hippocampus**. (A) A low magnification image of DGKβ immunoperoxidase in coronal section of the hippocampus from adult rat brain using guinea pig anti-DGKβ antibody. (B and C) High magnification images of DGKβ immunoperoxidase (B) and Nissl stain (C) in the hippocampus. (D) DGKβ is detected in fractionated protein samples from the hippocampus (Cyt, cytosolic fraction; LM, light membrane/microsome-enriched fraction; TSM, Triton-soluble synaptosomal membrane fraction; PSD, postsynaptic density fraction). DG, dentate gyrus; Or, stratum oriens; Py, pyramidal layer; Ra, stratum radiatum; Gr, granule cell layer. Scale bars, 500 μm (A); 100 μm (B).

In the hippocampal region, including the CA1, CA2, and CA3 subfields, the stratum oriens and stratum radiatum were generally more strongly immunolabeled with DGKβ antibody, while the dentate gyrus showed somewhat fainter immunolabeling (Figure 1A and 1B). In each of the labeled regions, immunohistochemical signals for DGKβ were seen as dense tiny puncta occupying the neuropil (Figure 1A and 1B).

Next, subcellular distribution was pursued biochemically by immunoblot with fractionated samples in the hippocampus. DGKβ was highly enriched in a light membrane/microsome-enriched fraction (LM), moderately in a postsynaptic density fraction (PSD), and faintly in a cytosolic (Cyt) and a Triton-soluble synaptosomal membrane fraction (TSM) (Fig 1D). Together with the immunohistochemical findings, the immunochemical analyses suggest that DGKβ is characterized by selective targeting to membranous and synaptic compartments.

### DGKβ is expressed in projection neurons and interneurons

**Figure 2:**
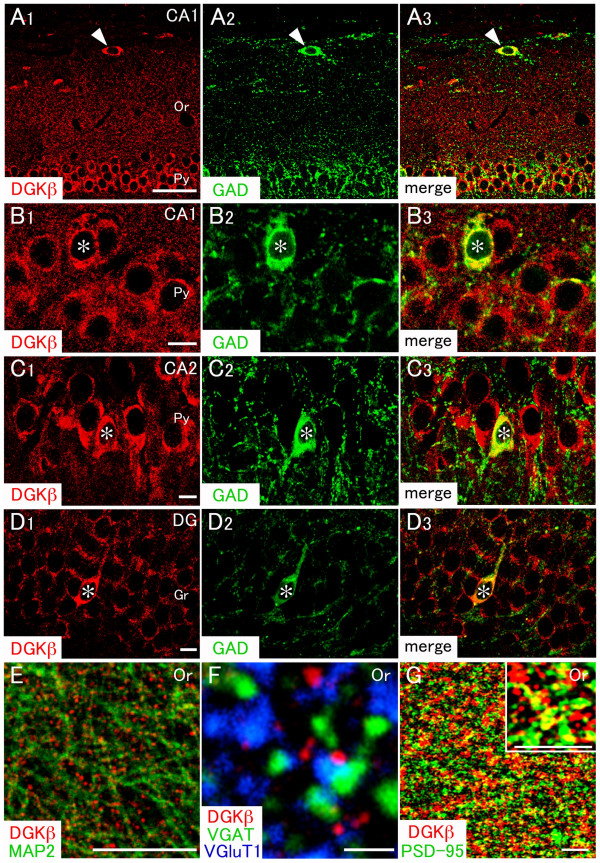
**Double immunofluorescence for characterization of DGKβ-expressing neurons in the hippocampus**. In all images, DGKβ is colored in red. Green fluorescence represents glutamate decarboxylase (A-D, GAD), microtubule associated protein-2 (E, MAP2), vesicular γ-aminobutylic acid transporter (F, VGAT), or postsynaptic density 95 kDa (G, PSD-95). Blue fluorescence represents vesicular glutamate transporter 1 (F, VGluT1). (A-D) DGKβ is co-expressed not only in scattered GAD-positive interneurons (*), but also in widely distributed GAD-negative projection neurons in the CA1, CA2, and dentate gyrus subfields. DGKβ is also detected in a GAD-positive non-pyramidal cell in the stratum oriens (A, arrowheads). (E) DGKβ is distributed on the surface of MAP-2-positive dendrites. (F and G) Punctate immunolabeling of DGKβ is not overlapped with that of presynaptic terminal markers, VGAT and VGluT1 (F), but partially with that of postsynaptic terminal marker, PSD-95 (G). Or, stratum oriens; Py, pyramidal layer; DG, dentate gyrus; Gr, granule cell layer. Scale bars, 50 μm (A); 10 μm (B-E); 1 μm (F); 4 μm (G).

Neurons in the hippocampus are mainly composed of two classes, including interneurons and projection neurons. The former can be distinguished by the expression of GAD, and the latter is GAD-negative [18]. Although immunoreactivities for both DGKβ and GAD were mainly distributed in the neuropil, the immunostaining successfully delineated somata expressing those proteins. Therefore we focused on the pyramidal layer, where cell bodies are located, to examine whether DGKβ is coexpressed in GAD-positive interneurons. When compared by double immunofluorescence, DGKβ was detected in both GAD-negative projection neurons and GAD-positive interneurons in the CA1, CA2, and dentate gyrus subfields (Figure 2A–D). In addition, a putative horizontal cell was labeled for DGKβ in the CA1 (arrowheads in Figure 2A). These results indicate that DGKβ is expressed not only in projection neurons but also in interneurons within the hippocampus.

### DGKβ is targeted preferentially to dendritic surface of neurons in the hippocampus

Subcellular localization of DGKβ was examined by double immunofluorescence using several subcellular markers (Figure 2E–G). When compared with somatodendritic neuronal marker MAP2, DGKβ-positive puncta were distributed along and around MAP2-positive dendritic shafts (Figure 2E). In triple immunofluorescent analysis, these DGKβ-positive puncta were apposed side by side to VGluT1-labeled excitatory and VGAT-labeled inhibitory terminals, but rarely overlapped with them (Figure 2F). In contrast, most of the DGKβ-positive puncta were overlapped with or continued to postsynaptic density protein PSD-95 (Figure 2G). These results suggest that DGKβ is selectively localized to postsynaptic elements. These features of DGKβ in hippocampal neurons were almost similar to those in the striatum [13].

### DGKβ is concentrated at perisynaptic sites of asymmetrical synapses

The postsynaptic membrane is a specialized receptive region composed of a dense molecular network, which often hinders antibody penetration and binding in conventional pre-embedding immunohistochemistry. This obstacle can be overcome by using post-embedding immunogold procedure, as has been demonstrated for ionotropic receptors and their scaffolding proteins in the postsynaptic density (PSD) [19-22] and also for other signaling molecules, such as phospholipase Cβ, and diacylglycerol lipase [22-25]. Therefore, we employed post-embedding immunogold technique to see whether DGKβ is particularly concentrated in the postsynaptic membrane and other membrane domains of hippocampal neurons.

**Figure 3:**
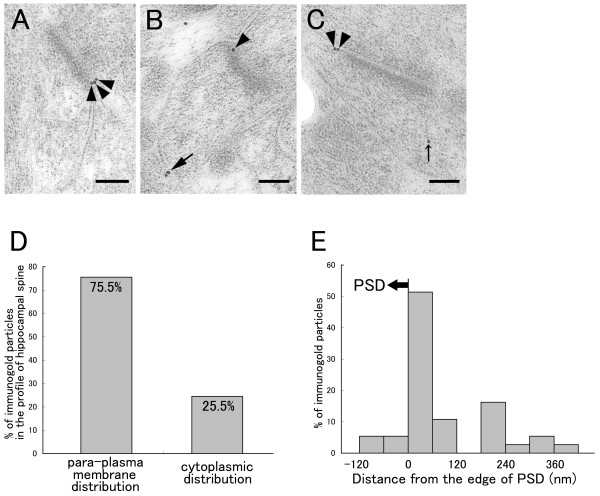
**Post-embedding immunoelectron microscopy for DGKβ in spines of hippocampal neurons**. (A-C) Electron micrographs showing immunogold localization of DGKβ in spines of hippocampal neurons. Arrowheads, large arrow, and small arrow indicate perisynaptic, extrasynaptic, or intracellular labeling, respectively. (D) The percentage of immunogold particles for cell membrane-associated (para-plasma membrane distribution) and intracellular (cytoplasmic distribution) labeling in spines of hippocampal neurons (n = 49 immunogold particles in 32 spines). Cell membrane-associated distribution was defined by the position of immunogold particles located less than 35 nm from the cell membrane to the center of gold particles. (E) A histogram showing tangential distribution of cell membrane-associated immunogold particles on spines of hippocampal neurons (n = 37 immunogold particles). DGKβ peaked in the 0–60-nm bin, which is just extrasynaptic from the edge of PSD. Scale bars, 100 nm.

In spines forming asymmetrical synapses of the hippocampus, most of the immunogold particles representing DGKβ were distributed close to the cell membrane, especially around the edges of synaptic junctions (arrowheads in Figure 3A–C). By taking the maximal length of immunoglobulins into consideration [26], we defined immunogold particles located less than 35 nm from the cell membrane to the center of gold particles as cell membrane-associated distribution. In spines forming asymmetrical synapses, 75.5% of immunogold particles were classified as cell membrane-associated distribution (Figure 3D). We further assessed the tangential distribution of cell membrane-associated gold particles by measuring the distance from the edge of PSD to the center of immunogold particles. The quantitative analysis showed that the distribution of gold particles was peaked at 0–60 nm bin from the edge of PSD (Figure 3E). The results from post-embedding immunogold method suggest that DGKβ accumulates on the perisynaptic site of asymmetrical synapses in the hippocampus.

### Developmental changes in the expression of endogenous DGKβ in cultured hippocampal neurons

**Figure 4:**
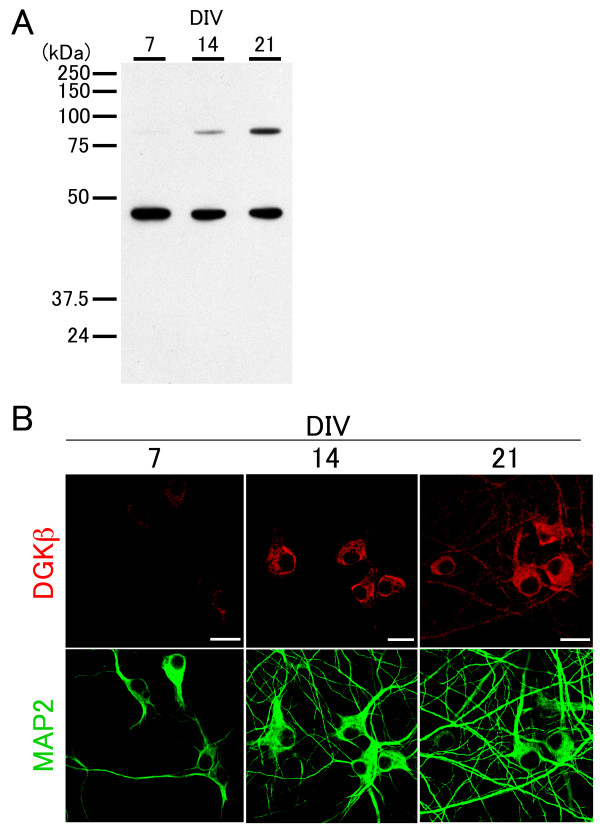
**Developmental changes in endogenous DGKβ expression in cultured hippocampal neurons**. (A) Western blots of DGKβ (upper bands; 90 kDa) in lysates from cultured neurons at 7, 14, and 21 DIV. β-actin was used as control (lower bands). (B) Immunocytochemistry for DGKβ in cultured neurons at 7, 14, and 21 DIV. MAP2 staining outlines developing dendrites in neurons. Scale bars, 20 μm.

It is reported at the organism level that the expression of DGKβ is undetectable at birth or on day 3 but rapidly increases between postnatal days 14 and 28, a stage of commencement of spinogenesis [12]. Therefore, we next examined the detailed expression and localization of DGKβ in developing hippocampal neurons in culture. We first ran Western blots to detect endogenous DGKβ in homogenized tissue from dissociated hippocampal cultures at 7–21 DIV (Figure 4A, top bands) and then performed immunocytochemical analysis (Figure 4B). Neurons in culture at 7 DIV contained few processes and hardly expressed DGKβ, while those at 14 DIV extended numerous processes although DGKβ was faintly expressed and was localized to the perinuclear region, but not in dendrites (Figure 4B). On the other hand, DGKβ greatly increased in the expression level in neurons at 21 DIV and was distributed throughout dendrites (Figure 4B). Together with the previous data at the organism level [12], these results indicate that DGKβ increases in the expression level and is distributed to dendrites at a later stage of dendritic development.

### DGKβ is involved in regulation of dendrite outgrowth and spine maturation

Relatively late onset of the expression and perisynaptic localization of DGKβ implicated its potential role in dendritic spine morphogenesis. It has been reported that dissociated hippocampal neurons rarely develop dendritic spines until 14 DIV [27]. Therefore, we next tested whether the overexpression of DGKβ could initiate spinogenesis early in the development. We focused on two stages of cultured neurons for this transfection experiment, i.e., 6 DIV, an immature stage of rare spine development, and 13 DIV, a stage of commencement of spine formation. At both stages, endogenous expression level of DGKβ was quite low, which was shown in Figure 4. In addition, our previous study revealed that DGKβ is targeted to certain, but not all, modalities of actin filaments in transfected COS-7 cells and is implicated in actin filament assembly through the enzymatic activity [15]. Therefore, we also asked if this is true for neurons.

**Figure 5:**
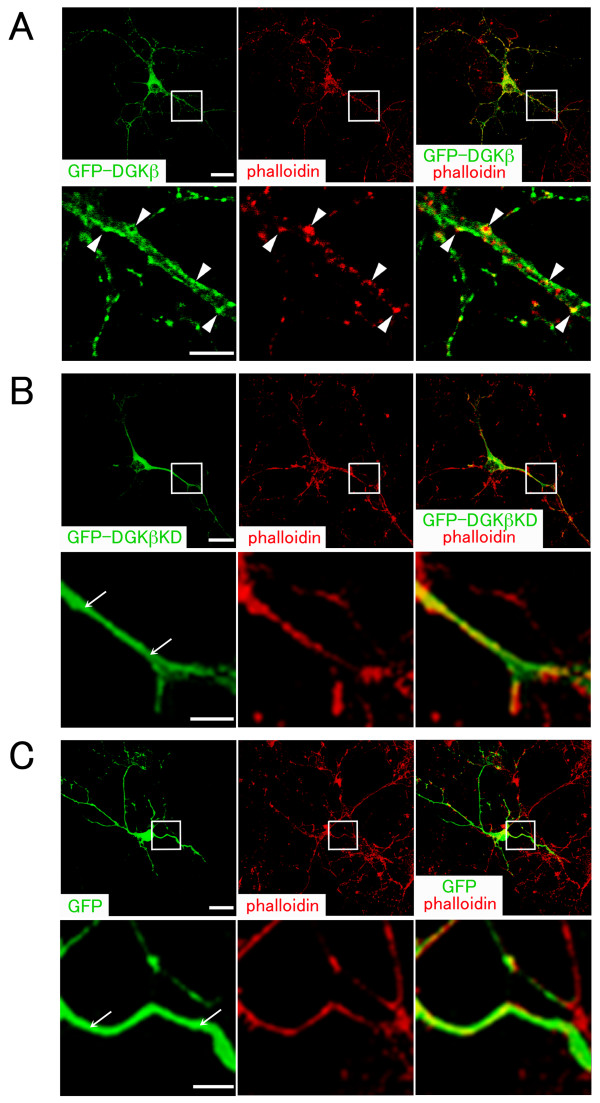
**Overexpression of DGKβ in hippocampal neurons at 6 DIV**. Cultured neurons were transfected at 6 DIV with GFP-fused wild-type DGKβ (GFP-DGKβ) (A), kinase-dead mutant of DGKβ (GFP-DGKβKD) (B), and control vector (GFP) (C). After 24 h incubation, they were fixed and stained with phalloidin to label F-actin (red). In each confocal image (A-C), boxed area is enlarged below. GFP-DGKβ is mostly observed along dendrites as puncta, which largely colocalize with clustered F-actin (arrowheads). On the other hand, GFP-DGKβKD and GFP show a diffuse pattern of localization (arrows). Scale bars, 25 μm (upper column in A-C); 5 μm (lower column in A-C).

To address this point, we transfected wild-type or kinase-dead mutant of DGKβ into cultured hippocampal neurons at 6 and 13 DIV and examined them after 24 h incubation. In hippocampal neurons transfected with wild-type DGKβ at 6 DIV, GFP-DGKβ roughly outlined the entire configuration of neurons and appeared as puncta of various sizes in dendrites (Figure 5A). It was mostly localized onto the plasma membrane in dendrites as a punctuated pattern. Phalloidin-labeled F-actin was also detected as puncta, which largely colocalized with GFP-DGKβ along dendrites (Figure 5A, arrowheads), although mature spines were never observed. On the other hand, kinase-dead mutant (GFP-DGKβKD) and GFP vector alone were observed in dendrites as a diffuse pattern (Figure 5B and 5C, arrows), which is clearly distinct from that of GFP-DGKβ.

**Figure 6:**
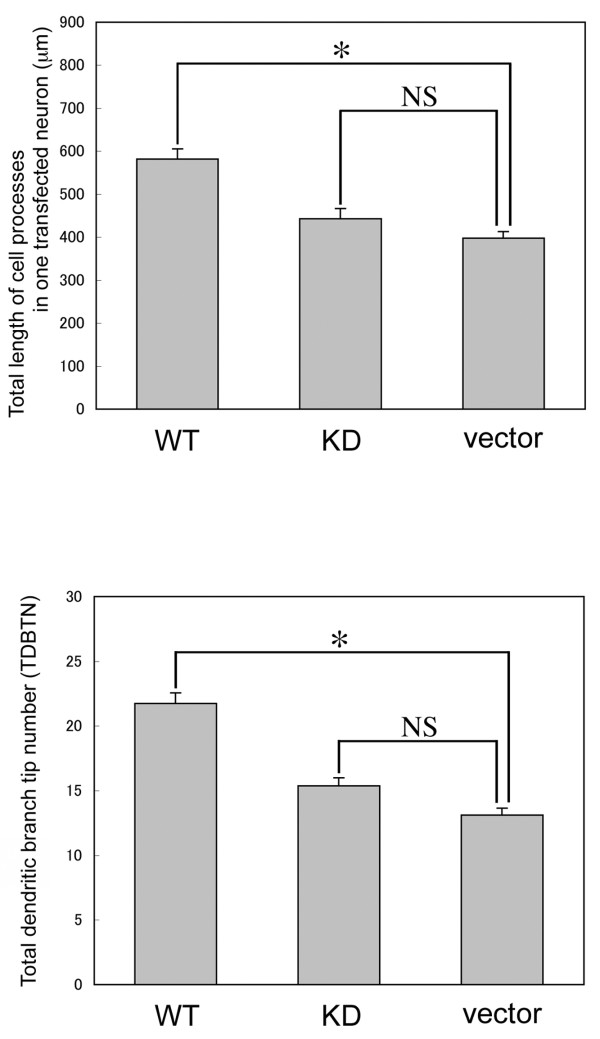
**Quantitative analysis of morphological changes in hippocampal neurons transfected at 6 DIV**. Histogram showing a quantitative analysis of total length of dendritic processes and TDBTN in neurons transfected with GFP-DGKβ (WT, n = 51 neurons), GFP-DGKβKD (KD, n = 45 neurons), and control vector (vector, n = 51 neurons) shown in Figure 5. The data are the mean ± S.E.M. *P < 0.01; NS, statistically not significant.

Neurons at this stage extended numerous cell processes, but spines were rarely recognized. When we measured total length of processes and total dendritic branch tip number (TDBTN) between neurons transfected with wild-type, KD mutant, or vector alone, it was revealed that total length of the cell processes and TDBTN of neurons transfected with wild-type DGKβ were greatly increased compared with those of neurons transfected with KD mutant or vector alone (Figure 6). Taken together, the transfection experiment at 6 DIV demonstrates the following: (1) Wild-type DGKβ induces clustering of F-actin along dendrites, although it cannot initiate spinogenesis by overexpression alone at this stage; (2) Wild-type DGKβ promotes dendritic outgrowth and branching; (3) The enzymatic activity of DGKβ has some effects on its own subcellular localization.

**Figure 7:**
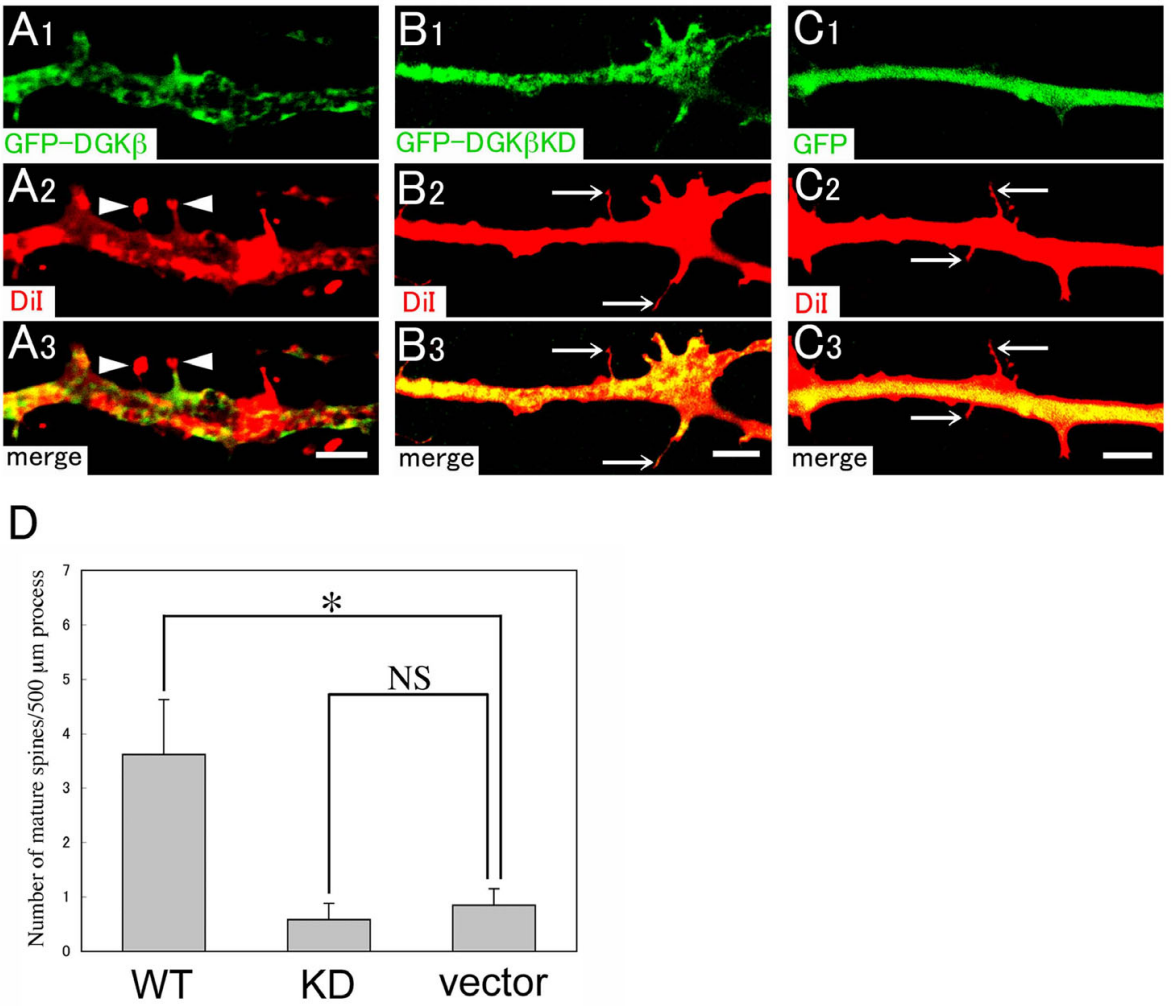
**Overexpression of DGKβ in hippocampal neurons at 13 DIV**. Cultured neurons were transfected at 13 DIV with GFP-DGKβ (A), GFP-DGKβKD (B), and control vector (GFP) (C). After 24 h incubation, they were fixed and stained with Vybrant-DiI (red) to outline the shape of dendritic spines. Note that mature spines of mushroom-type (A, arrowheads) are frequently observed in neurons transfected with GFP-DGKβ, but hardly with GFP-DGKβKD (B, arrows) and control vector (C, arrows). (D) Histogram showing the density of mature spines per 500 μm dendrite in neurons transfected with GFP-DGKβ (n = 5 neurons), GFP-DGKβKD (n = 7 neurons), and control vector (n = 6 neurons). The data are the mean ± S.E.M. *P < 0.05; NS, statistically not significant. Scale bars, 5 μm.

On the other hand, hippocampal neurons at 14 DIV contained more and longer processes compared with those at 7 DIV (Figure 4B). In addition, numerous immature spines with long and thin stalk (headless spine) were recognized in untransfected neurons, although mature spines of mushroom type were very rarely observed. There were no changes in the number of dendritic protrusion between neurons transfected with wild-type, KD mutant, or vector alone (data not shown). However, we found a significant increase in the number of mature spines of the mushroom-type in neurons transfected wild-type DGKβ (Figure 7A and 7D), while mature spines were hardly observed in neurons transfected with KD mutant or vector alone (Figure 7B–D). These data suggest that DGKβ promotes spine maturation through the enzymatic activity in dendritic spine morphogenesis.

## Discussion

DGKβ was first identified as an isozyme expressed in neuronal population in the brain [11]. The characteristic gene expression in the brain, i.e., predominant expression in the striatum, provoked us to perform a detailed investigation of its localization, which reveals that DGKβ is expressed in medium spiny neurons constituting the striatonigral and striatopallidal pathways and exhibits dense accumulation at perisynaptic sites on dendritic spines forming asymmetrical synapses [13]. In the present study, we investigated the functional implications of DGKβ using cultured hippocampal neurons transfected with wild-type and kinase-dead mutant of DGKβ. We found that DGKβ shows similar characteristics in its subcellular localization in hippocampal neurons, i.e., predominant localization on perisynaptic membrane, and that DGKβ promotes dendritic outgrowth and spine maturation in transfected neurons through the enzymatic activity.

How does the enzymatic activity of DGKβ regulate the dendrite outgrowth and spine maturation in developing neurons? Although we are short of experimental evidence, it is speculated that the substrate and/or product of DGK, i.e. DG and PA, might play key roles in these phenomena. It has been shown that the metabolism of PI including PA is implicated in the regulation of actin cytoskeleton assembly [28,29]. One possibility is that PA, produced by DGKβ at the perisynaptic membrane, may regulate the remodeling system via actin filaments. Dendritic spines show remarkable morphological diversity and plasticity, presumably due to dynamic alterations of the underlying actin cytoskeleton [16]. Dendritic spines are mushroom-like, membranous protrusions from dendrites and are important in normal cognitive function and neuronal development [17,30]. The morphology of spines is a strong determinant of their function, and the actin cytoskeleton is believed to be the basic structural foundation that is responsible for the shape [17].

The RhoGTPase family, including RhoA, cdc42, and Rac1, modulates the actin cytoskeleton in fibroblasts, budding yeast, and neurons and is thought to be the major regulators of spine morphogenesis [31-35]. Previous study has shown that Rac1 can initiate spinogenesis by overexpression alone in cultured neurons [36]. Rac1 induces the formation of dendritic spines in neurons as young as 7 DIV and clusters in the heads of dendritic spines. Furthermore, Rac1 increases the size of preexisting spines in mature neurons (>21 DIV) within 24 h after transfection. Rac1 is also shown to regulate actin polymerization [37], and actin is essential for the anchoring of postsynaptic receptors such as NMDA, AMPA, and GABA receptors in mature spines [38]. These studies suggest that Rac1 is deeply involved in spinogenesis through the control of actin polymerization. Taken the present and previous data together, DGKβ cannot initiate spinogenesis at 6 DIV by the overexpression alone even though it can induce clustering of F-actin along dendrites (Figure 5A), while Rac 1 can initiate spinogenesis at this stage. We hypothesize that DGKβ plays a role in clustering of F-actin and represents an upstream effector of Rac 1 in spinogenesis. Based on this hypothesis, inability of spinogenesis by DGKβ alone at 6 DIV may be explained by lack of a putative downstream effector Rac 1 expression at this stage. This is the major issue to be addressed in our future study.

With regard to the molecular mechanism, it is shown that the C-terminus of Rac1 is associated specifically with type I PI(4)P5-kinase [39]. Intriguingly, Rac is also shown to be associated with DGK and RhoGDI, although it remains to be determined which DGK isozyme(s) is responsible for the association with Rac. In this signaling complex it may be that DGK generates PA, which acts as a strong activator of PI(4)P5-kinase [6], thus increasing production of PIP2. Therefore, it seems that Rac brings PI(4)P5-kinase and PA into close proximity, thus increasing the production of PIP2, which binds to, and regulates, several actin regulatory proteins, including gelsolin, profilin, α-actinin, and capZ [28,40]. What is the functional role of RhoGDI in this complex? It is hypothesized that once released from RhoGDI the Rac-lipid kinase complex is stabilized by phospholipids, such as PIP2 and PA, at the membrane, suggesting that RhoGDI might control the subcellular localization of this complex. Inability of kinase dead DGKβ to localize to the correct sites, but staying in the cytoplasm, as shown in Figure 5B, may be due to the decreased production of PA, which leads to the reduced level of PIP2 and subsequent lowered stabilization of the complex to the membrane.

It is reported that the human DGKβ gene can generate several enzyme isoforms [14]. In that study, the wild type and the splice variant lacking 35 amino acid C-terminal region (SV3'DGKβ) show different subcellular localization in cDNA-transfected HEK293 cells, although they exhibit similar catalytic activity [14]. This raises the additional possibility that C-terminal region, as well as the catalytic activity, might also influence subcellular localization of DGKβ. In terms of pathophysiological implication, it is noteworthy that SV3'DGKβ is annotated in GenBank as being differentially expressed in bipolar disorder patients [14]. Considering that DGKβ is abundantly expressed in the striatum, accumbens nucleus, and hippocampus [11], changes in the activity and/or subcellular localization of DGKβ should influence the signaling cascade in these regions that are known to be involved in the control of emotional and cognitive behavior.

Dendritic spine dysgenesis may also be related to psychiatric diseases, such as mood disorders (schizophrenia and bipolar disorder) and depressive conditions [41-47]. People affected by these disorders and conditions show a variety of symptoms, which may be ascribed to the brain areas afflicted by signal dysfunction. Considering these, the impairment of spine formation due to DGKβ dysfunction predominantly in the striatum could lead to the impaired signaling in neurons of this area. Increased or decreased expression of DGKβ and its splice variants and/or altered subcellular localization could impair a remodeling process of spines. As DGKβ seems to be involved in actin-based cytoskeletal process, dysfunction of DGKβ due to the alteration of catalytic activity and subcellular localization may disturb actin filament system in the process of spine formation. Our previous study supports this possibility because overexpression of DGKβ causes altered assembly of actin stress fibers in transfected COS-7 cells [15]. In addition, the kinase-dead mutant localizes diffusely to the cytoplasm, suggesting that the catalytic activity of DGKβ affects its own localization. The phenomena observed in transfected COS-7 cells are now reproduced in neurons in a similar, but not identical, fashion. It is unclear whether the colocalization of DGKβ with F-actin depends on their direct/indirect association. In this respect, our previous study revealed in transfected COS-7 cells that DGKβ is colocalized with actin stress fibers, but not with actin bundles in pseudopodia at the periphery [15]. These data suggest the possibility that DGKβ is not bound to actin fibers directly but through actin-binding protein(s).

It remains elusive why DGKβ shows distinct expression patterns between the hippocampus and striatum. DGKβ is expressed not only in projection neurons but also in interneurons in the hippocampus, while it is solely expressed in projection neurons in the striatum [13]. In this regard, previous studies have shown that distinct subtypes of the mGluR family are expressed in different subsets of neurons, i.e., mGluR5 is abundantly expressed in projection neurons, while mGluR1α is dominant in interneurons in the striatum [48-50]. In the hippocampus, on the other hand, mGluR5 is highly expressed in pyramidal neurons including projection neurons [51,52] and parvalbumin-positive interneurons [49,53]. These features of the mGluR5 expression pattern in the striatum and hippocampus seem to be similar to that of DGKβ, suggesting that DGKβ and mGluR5 work cooperatively in the same complex for signal transduction machinery.

In furure studies, we need to address several points discussed above and to ask whether roles played by DGKβ and other DGKs, including DGKγ, -ε, -ζ, and -ι, are equivalent or distinct in neurons. Detailed investigation of the relationship between DGKs and the related molecules in spinogenesis is currently underway.

## Conclusion

In this study we found that DGKβ exists not only in projection neurons but also in interneurons in the hippocampus and is abundant at perisynaptic sites. In addition, we show for the first time that overexpression of wild-type DGKβ induces dendrite outgrowth and spine maturation in developing hippocampal neurons through its enzymatic activity, suggesting that DGKβ is involved in these molecular machineries. These findings would help understand the pathophysiological mechanisms of spinogenesis and synaptic plasticity.

## Methods

### Tissue and section preparation

This study was carried out in accordance with Guide for Animal Experimentation, Yamagata University School of Medicine. Adult Wistar rats at 9 weeks of age (Japan SLC) were used in the present study. For immunohistochemistry, rats anesthetized with ether were fixed transcardially with 4% paraformaldehyde in 0.1 M sodium phosphate buffer (pH 7.2) for light and immunofluorescence microscopies or 4% paraformaldehyde/0.1% glutaraldehyde in 0.1 M sodium phosphate buffer (pH 7.2) for immunoelectron microscopy. Paraffin sections (5 μm in thickness) were prepared for immunoperoxidase by a sliding microtome (SM2000R; Leica, Nussloch, Germany), while microslicer sections (50 μm; VT1200S, Leica) were prepared for immunofluorescence microscopy. For post-embedding immunogold electron microscopy, microslicer sections (400 μm) were prepared.

### Immunoblotting

Biochemical subcellular fractionation was performed as reported previously [13,23]. The hippocampi of adult Wistar rats, anesthetized with ether, were rapidly removed from the skull and homogenized using a Potter homogenizer with 15 strokes at 800 r.p.m. in 4 volumes of ice-cold homogenize buffer containing 0.32 M sucrose, 1 mM EDTA, 1 mM EGTA, 10 mM Tris-HCl (pH 7.0) and 0.4 mM phenylmethylsulfonyl fluoride (PMSF). The homogenate was centrifuged at 1000 g for 10 min to remove nuclei and large debris. The supernatant (S1, postnuclear fraction) was centrifuged at 10000 g for 20 min to obtain a crude synaptosomal fraction, lysed hypo-osmotically and centrifuged at 25000 g for 30 min. The pellet (synaptosomal membrane) was suspended with 0.5% Triton X-100 in the homogenize buffer for 15 min and centrifuged at 111000 g for 1 h to separate a postsynaptic density fraction (PSD, pellet) and a Triton-soluble synaptosomal membrane fraction (TSM, supernatant). The supernatant after centrifugation of S1 was further centrifuged at 165000 g for 1 h to obtain a cytosolic fraction (Cyt) and a light membrane/microsome-enriched fraction (LM). The protein concentration was determined by BCA protein assay kit (Pierce, Rockford, IL). Proteins were separated on 10% sodium dodecyl sulphate-polyacrylamide gel by electrophoresis, and then electroblotted onto a polyvinylidene difluoride (PVDF) membrane (NENTM Life Science Products, Inc., Boston, MA). After blocking with 5% non-fat dry milk (w/v) in PBS containing 0.02% sodium azide and 0.2% Tween 20 for 1 h, membranes were incubated for 1 h at room temperature with guinea pig anti-DGKβ antibody (0.5 μg/ml) [13] diluted with PBS containing 0.1% Tween 20 and then with peroxidase-linked secondary antibody (1:10000, GE Healthcare UK Ltd, Buckinghamshire, England) for 30 min. Immunoreaction was visualized with the chemiluminescent ECL Plus Western blotting detection system (GE Healthcare UK Ltd).

Cultured dissociated hippocampal neurons at 7, 14, or 21 DIV were freeze-thawed and disrupted by sonication in lysis buffer containing 50 mM Tris-HCl (pH 7.4), 150 mM NaCl, 1 mM PMSF, and an appropriate amount of protease inhibitor cocktail (Complete; Roche Diagnostics GmbH, Mannheim, Germany). Total homogenate was cleaned by centrifugation at 3000 g for 10 min to remove cell debris. The resulting supernatant was boiled for 5 min in SDS sample buffer. The proteins were then electrophoretically transferred to a PVDF membrane. After blocking the non-specific binding sites with 5% non-fat dry milk (w/v) in PBS containing 0.02% sodium azide and 0.2% Tween 20 for 1 h, the membrane was incubated for 1 h at room temperature with guinea pig anti-DGKβ antibody (0.5 μg/ml) [13] and mouse anti-β-actin (1: 5000; A5441; Sigma-Aldrich, Saint Louis, MO) in PBS containing 0.1% Tween 20. Sites of antigen-antibody reaction were visualized using the chemiluminescent ECL Plus Western blotting detection system (GE Healthcare UK Ltd).

### Primary hippocampal culture

Primary cultured hippocampal neurons were prepared from embryonic day 18 (E18) or E19 Wistar rats under deep anesthesia with diethyl ether as described previously [54,55]. Dissociated cells were plated onto poly-D-lysine-coated plastic dishes in a growth medium consisting of Neurobasal medium (Invitrogen, Carlsbad, CA) supplemented with B27 supplement (Invitrogen) and 0.25 mM L-glutamine at a density of 1.0 × 10^6 ^cells per 60-mm dish, and maintained in a humidified incubator with 5% CO2 at 37°C. One-half of the medium was changed every 5 days after plating. At 7, 14, and 21 DIV, the cells were fixed with 4% paraformaldehyde in 0.1 M sodium phosphate buffer (pH 7.2) for 10 min and were treated with 100% methanol for 5 min. After blocking in 10% normal goat serum, the cells were incubated with mouse anti-microtubule associated protein-2 (MAP2; 1:500; MAB378; Chemicon, Temecula, CA) together with guinea pig anti-DGKβ (1.5 μg/ml) [13] overnight at room temperature (~20°C). They were subsequently incubated with Alexa 546-conjugated anti-guinea pig IgG (Molecular Probes, Inc, Eugene, OR) and Alexa 488-conjugated anti-mouse IgG (Molecular Probes, Inc.). The immunoreaction was observed by a confocal laser scanning microscopy (LSM510META, Carl Zeiss, Göttingen, Germany).

### Transfection of primary rat hippocampal neurons and immunostaining

Wild-type and kinase-dead muntant of rat DGKβ were transfected by Lipofectamine 2000 (Invitrogen) into cultured hippocampal neurons at 6 and 13 DIV [54]. Kinase-dead mutant of DGKβ (DGKβKD) was generated replacing in the ATP binding domain GxGxxG with GxDxxG [56] using the Quick-change Site-directed Mutanegesis Kit (Stratagene, La Jolla, CA); G495D for DGKβ [15]. The cDNAs for wild-type and kinase-dead mutant of DGKβ were subcloned in the expression vector pEGFP-C2 (CLONTECH Laboratories, Inc., Palo Alto, CA). All of the expression vectors for neuronal transfection were purified by using EndoFree Plasmid Maxi kit (Qiagen, Germantown, MD). After 24 h incubation, hippocampal neurons transfected with DGKβ/pEGFP, DGKβKD/pEGFP, or pEGFP were fixed with 4% paraformaldehyde in 0.1 M sodium phosphate buffer (pH 7.2) for 15 min at room temperature. After washing in PBS, neurons transfected at 6 DIV were incubated with Alexa Fluor 568-phalloidin (1:40, A12380, Molecular Probes, Inc.) for 1 h at room temperature and those at 13 DIV were incubated with Vybrant-DiI cell-labeling solution (1: 200, V22885, Molecular Probes) overnight at room temperature. To identify transfected cells as neurons, we also used anti-MAP2 mouse monoclonal IgG (1:500; MAB378; Chemicon) and Alexa 647-conjugated anti-mouse IgG (Molecular Probes, Inc). Fluorescent images were viewed with confocal laser scanning microscope (LSM510META, Carl Zeiss).

### Image analysis

For quantification of dendritic morphology, total dendritic length and total dendritic branch tip number (TDBTN) were measured as described previously [57-59], using ImageJ software. MAP2-positive transfected neurons were collected per construct from two independent experiments. Dendritic tips were scored when they were longer than 3 μm as described previously [57,59]. For quantification of the density of dendritic spines, a dendritic protrusion with an expanded head that was 50% wider than its neck was defined as a spine [36]. The number of spines from one neuron was counted manually and normalized per 500 μm dendritic length. Statistical difference was determined by Mann-Whitney U test.

### Immunohistochemistry

Immunohistochemical analyses were performed as described previously [13]. Microslicer sections were dipped successively in 30, 60, and 100% methanol for 2 min each before incubation with primary antibodies. All immunohistochemical incubations were performed at room temperature. Immunoperoxidase staining on paraffin sections was performed by overnight incubation with guinea pig anti-DGKβ antibody (0.5 μg/ml) [13]. Sections were further incubated with biotinylated secondary antibodies (Vector Laboratories, Burlingame, CA) for 30 min and avidin-biotin-peroxidase complex for 30 min using the avidin-biotinylated peroxidase complex (ABC) system (Vector Laboratories). Immunoreaction was visualized with 3,3-diaminobenzidine (DAB) and photographs were taken by an microscope (Leica). In double immunofluorescence, rabbit or guinea pig anti-DGKβ antibodies [13] diluted to 1.5 μg/ml with PBS containing 0.1% Triton X-100 was immunoreacted overnight in a mixture with one of the following antibodies: rabbit anti-glutamate decarboxylase (GAD; 1:2000; AB5992; Chemicon), mouse anti-MAP2 (1:100; MAB3418; Chemicon), goat anti-vesicular glutamate transporter 1 (VGluT1; 1 μg/ml) [60], guinea pig anti-vesicular γ-aminobutylic acid transporter (VGAT; 1 μg/ml) [60], mouse anti-postsynaptic density 95 kDa (PSD-95; 2 μg/ml; MA1-045, Affinity BioReagents, Inc., Golden, CO). They were visualized by 2 h incubation with species-specific secondary antibodies at a dilution of 1: 200 (Molecular Probes, Inc.). Images were taken with a confocal laser scanning microscope (LSM510META, Carl Zeiss).

For post-embedding immunogold method, hippocampal slices were cryoprotected with 30% sucrose in 0.1 M sodium phosphate buffer and frozen rapidly with liquid propane in an EM CPC unit (Leica). Frozen sections were immersed in 0.5% uranyl acetate in methanol at -90°C in an AFS freeze-substitution unit (Leica), infiltrated at -45°C with Lowicryl HM-20 resin (Lowi, Waldkraiburg, Germany) and polymerized with UV light. After etching with saturated sodium-ethanolate solution for 3 s, ultra-thin sections on nickel grids were treated successively with 1% human serum albumin (Wako, Osaka, Japan)/0.1% Triton X-100 in Tris-buffered saline (pH 7.5) (TBS-T) for 1 h, rabbit anti-DGKβ antibody (20 μg/ml) [13] in 1% human serum albumin/TBS-T overnight and colloidal gold (10 nm)-conjugated anti-rabbit IgG (1:100; British Bio Cell International, Cardiff, UK) in 1% human serum albumin/TBS-T for 2 h. Finally, grids were stained with uranyl acetate for 15 min and examined with an H-7100 electron microscope (Hitachi, Tokyo, Japan). Quantitative analysis was performed using two animals for post-embedding immunogold methods as above. Metal particles were counted on electron micrographs and analyzed using iTEM software (Olympus, Tokyo, Japan). Perpendicular distribution of post-embedding immunogold particles from the cell membrane was analyzed in spines. The cell membrane was indicated as 0 nm, and the distance from the cell membrane to gold particle was measured. Cell membrane-associated distribution was defined by the position of immunogold particles located less than 35 nm from the cell membrane to the center of gold particles [23]. Tangential distribution of post-embedding immunogold particles from PSD was analyzed in the spine of hippocampal cells. The edge of PSD was indicated as 0 nm, and we measured the distance from the edge of PSD to gold particles, which were clearly associated to the cell membrane. For the evaluation of perisynaptic localization of DGKβ, the distance from the edge of PSD was divided into 60-nm segments [13,23]. The ordinate indicates the percentage of gold particles in each 60-nm segment. We observed 2 particles in sections treated with antigen-pre-absorbed antibody in 32 spines.

## Abbreviations

DG: diacylglycerol; DGK: diacylglycerol kinase; DIV: day in vitro; EST: Expressed Sequence Tag; GAD: glutamate decarboxylase; GFP: green fluorescent protein; IP3: inositol 1,4,5-trisphosphate; mGluR: metabotropic glutamate receptor; KD: kinase-dead; MSNs: medium spiny neurons; PI: phosphoinositide; PA: phosphatidic acid; PBS: phosphate-buffered saline; PLC: phospholipase C; PSD: postsynaptic density; VGAT: vesicular γ-aminobutylic acid transporter; VGluT1: vesicular glutamate transporter 1.

## Authors' contributions

YH and KG designed research. YH performed research and analyzed data. MW and KO contributed to the intellectual progression of the study. YH and KG wrote the paper. All authors read and approved the final manuscript.
